# Altered Capicua expression drives regional Purkinje neuron vulnerability through ion channel gene dysregulation in spinocerebellar ataxia type 1

**DOI:** 10.1093/hmg/ddaa212

**Published:** 2020-09-23

**Authors:** Ravi Chopra, David D Bushart, John P Cooper, Dhananjay Yellajoshyula, Logan M Morrison, Haoran Huang, Hillary P Handler, Luke J Man, Warunee Dansithong, Daniel R Scoles, Stefan M Pulst, Harry T Orr, Vikram G Shakkottai

**Affiliations:** Medical Scientist Training Program, University of Michigan Medical School, Ann Arbor, MI 48109, USA; Department of Neurology, University of Michigan Medical School, Ann Arbor, MI 48109, USA; Department of Neurology, Washington University in St. Louis, Saint Louis, MO 63110, USA; Department of Neurology, University of Michigan Medical School, Ann Arbor, MI 48109, USA; Department of Molecular and Integrative Physiology, University of Michigan, Ann Arbor, MI 48109, USA; Ohio State University College of Medicine, Columbus, OH 43210, USA; Department of Neurology, University of Michigan Medical School, Ann Arbor, MI 48109, USA; Department of Molecular Biosciences and Institute for Cellular and Molecular Biology, University of Texas at Austin, Austin, TX 78712, USA; Department of Neurology, University of Michigan Medical School, Ann Arbor, MI 48109, USA; Department of Neurology, University of Michigan Medical School, Ann Arbor, MI 48109, USA; Department of Neurology, University of Michigan Medical School, Ann Arbor, MI 48109, USA; Department of Laboratory Medicine and Pathology, Institute for Translational Neuroscience, University of Minnesota, Minneapolis, MN 55455, USA; Department of Neurology, University of Michigan Medical School, Ann Arbor, MI 48109, USA; Department of Neurology, University of Utah, Salt Lake City, UT 84132, USA; Department of Neurology, University of Utah, Salt Lake City, UT 84132, USA; Department of Neurology, University of Utah, Salt Lake City, UT 84132, USA; Department of Laboratory Medicine and Pathology, Institute for Translational Neuroscience, University of Minnesota, Minneapolis, MN 55455, USA; Department of Neurology, University of Michigan Medical School, Ann Arbor, MI 48109, USA; Department of Molecular and Integrative Physiology, University of Michigan, Ann Arbor, MI 48109, USA

## Abstract

Selective neuronal vulnerability in neurodegenerative disease is poorly understood. Using the ATXN1[82Q] model of spinocerebellar ataxia type 1 (SCA1), we explored the hypothesis that regional differences in Purkinje neuron degeneration could provide novel insights into selective vulnerability. ATXN1[82Q] Purkinje neurons from the anterior cerebellum were found to degenerate earlier than those from the nodular zone, and this early degeneration was associated with selective dysregulation of ion channel transcripts and altered Purkinje neuron spiking. Efforts to understand the basis for selective dysregulation of channel transcripts revealed modestly increased expression of the ATXN1 co-repressor Capicua (Cic) in anterior cerebellar Purkinje neurons. Importantly, disrupting the association between ATXN1 and Cic rescued the levels of these ion channel transcripts, and lentiviral overexpression of Cic in the nodular zone accelerated both aberrant Purkinje neuron spiking and neurodegeneration. These findings reinforce the central role for Cic in SCA1 cerebellar pathophysiology and suggest that only modest reductions in Cic are needed to have profound therapeutic impact in SCA1.

## Introduction

The polyglutamine disorders are a class of autosomal dominant neurodegenerative diseases caused by a glutamine-encoding CAG triplet repeat (polyglutamine) expansion within the protein coding sequence for their respective disease gene. These disorders are characterized by neurodegeneration in a restricted subset of neuron types despite widespread pathogenic protein expression, a phenomenon often referred to as ‘selective vulnerability’. The importance of understanding selective vulnerability as a strategy for uncovering fundamental neurodegenerative disease pathways is widely acknowledged (1,2), but mechanisms of selective vulnerability remain poorly understood.

Spinocerebellar ataxia type 1 (SCA1) is a polyglutamine disorder caused by CAG repeat expansion within the Ataxin-1 (*ATXN1*) gene (3). Like other polyglutamine ataxias, SCA1 is characterized by predominant involvement of cerebellar and brainstem neurons (4) despite widespread CNS expression of ATXN1 protein (5). Cerebellar Purkinje neuron degeneration is prominent in SCA1 (6), suggesting that Purkinje neurons are particularly vulnerable to the expression of polyglutamine-expanded ATXN1. This conclusion is supported by a transgenic model of SCA1 expressing ATXN1 with 82 CAG repeats (ATXN1[82Q]) restricted to Purkinje neurons, as this mouse has prominent Purkinje neuron degeneration and motor impairment (7). Further study of this model has made it clear that dysregulation of gene expression is central to SCA1 pathogenesis (8,9), but the key disease-associated genes underlying Purkinje neuron degeneration are not well understood.

Relative sparing of Purkinje neurons in lobule X of the ATXN1[82Q] model has been noted (10), despite the fact that ATXN1[82Q] expression is driven by a promoter expected to produce widespread Purkinje neuron expression (11). This finding suggests that Purkinje neurons in the anterior cerebellum are particularly vulnerable to the ATXN1[82Q] expression. Recognizing that efforts to understand selective vulnerability are confounded by the numerous differences between affected and unaffected cell types, we proposed that regional differences in Purkinje neuron degeneration could reveal novel pathways central to disease.

In the current study, we explored the hypothesis that there are specific disease-associated genes whose regional pattern of dysregulation matches the regional pattern of Purkinje neuron degeneration. We demonstrated robust confirmation of prior qualitative observations that Purkinje neuron degeneration is accelerated in the anterior cerebellum (lobules II–V) relative to the nodular zone (lobules IX–X). Consistent with our hypothesis, we found that not all disease-associated genes are dysregulated in a manner that correlates with this degeneration pattern. Instead, only a subset of disease-associated ion channel genes is dysregulated exclusively in anterior Purkinje neurons, and selective anterior Purkinje neuron firing abnormalities are observed that can be well-explained by changes in the affected channels. We identified modestly higher expression of the ATXN1 co-repressor, Capicua (Cic), in the anterior cerebellum in association with this regional ion channel gene suppression. Finally, we found that dysregulation of disease-associated ion channel genes was Cic-dependent, as disrupting the association between ATXN1 and Cic increased the levels of these ion channel transcripts, while viral overexpression of Cic in the nodular zone recapitulated the firing abnormalities seen in the anterior cerebellum and also accelerated nodular zone neurodegeneration. Our findings underscore the central role for Cic in SCA1 cerebellar pathophysiology (9). These findings provide important insights regarding Purkinje neuron vulnerability in SCA1 and elevate these channels as important targets for the development of disease-modifying therapy.

## Results

### Purkinje neurons from the anterior cerebellum are more vulnerable than Purkinje neurons from the nodular zone in SCA1 mice

**Figure 1:**
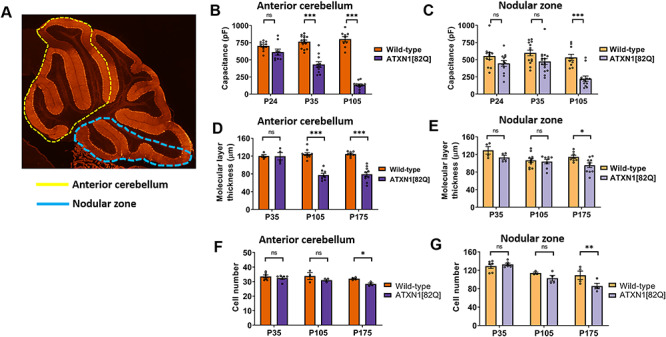
Purkinje neuron degeneration is delayed in the nodular zone of ATXN1[82Q] mice. (**A**) Diagram outlining the anterior cerebellar lobules (yellow dotted line) and nodular zone (blue dotted line). (**B** and **C**) Total Purkinje neuron capacitance was measured in the anterior cerebellum (B) and nodular zone (C) of ATXN1[82Q] mice and wild-type controls at P24, P35 and P105. (**D** and **E**) Molecular layer thickness, a measurement which reflects the length of Purkinje neuron dendrites, was measured in the anterior cerebellum (D) and nodular zone (E) of ATXN1[82Q] mice and wild-type controls at P35, P105 and P175. (**F** and **G**) Cell number was measured in the anterior cerebellum (F) and nodular zone (G) of ATXN1[82Q] mice and wild-type controls at P35, P105 and P175. *Denotes *P* < 0.05, ***denotes *P* < 0.001, ns denotes *P* > 0.05; two-way repeated-measures ANOVA with Holm-Sidak correction for multiple comparisons.

We began by quantifying the kinetics of neurodegeneration in Purkinje neurons from anterior cerebellum and compared them to Purkinje neurons from the nodular zone (Fig. 1A, Supplementary Material, Fig. S1A and B), as previous studies have noted relative sparing of Purkinje neurons in lobule X found within the nodular zone (10). Measurement of membrane capacitance (Fig. 1B and C, Supplementary Material, Fig. S1C–F) and molecular layer thickness (Fig. 1D and E) showed that dendritic degeneration begins earlier in the anterior cerebellum of ATXN1[82Q] mice, first detected by capacitance measurement in anterior cerebellum by P35. Cell loss is identified at P175 in both the anterior cerebellum and nodular zone in ATXN1[82Q] mice (Fig. 1F and G). The early onset of Purkinje neuron degeneration starting at P35 is not explained by differences in ATXN1[82Q] transgene expression at that age (Supplementary Material, Fig. S1G), though there is reduced transgene expression at P105 (Supplementary Material, Fig. S1H). These findings suggest that Purkinje neurons from the anterior cerebellum are more vulnerable in ATXN1[82Q] mice and argue for the existence of a disease pathway unique to anterior Purkinje neurons in the ATXN1[82Q] model of SCA1.

### A functionally related module of channels is dysregulated uniquely in the anterior cerebellum of SCA1 mice

Polyglutamine-expanded ATXN1 is thought to exert its pathogenic effect in Purkinje neurons primarily by disrupting gene expression. Dysregulation of transcripts and transcript modules has been suggested as the root cause for disease (12), and unbiased analysis of ataxia genes demonstrates that these genes fit within a limited number of pathways to unify Purkinje neuron vulnerability across spinocerebellar ataxias (13). Altered Purkinje neuron intrinsic excitability has emerged as one particularly important pathway (14), suggesting that there may be a limited number of channels that unify Purkinje neuron dysfunction across ataxias.

**Figure 2:**
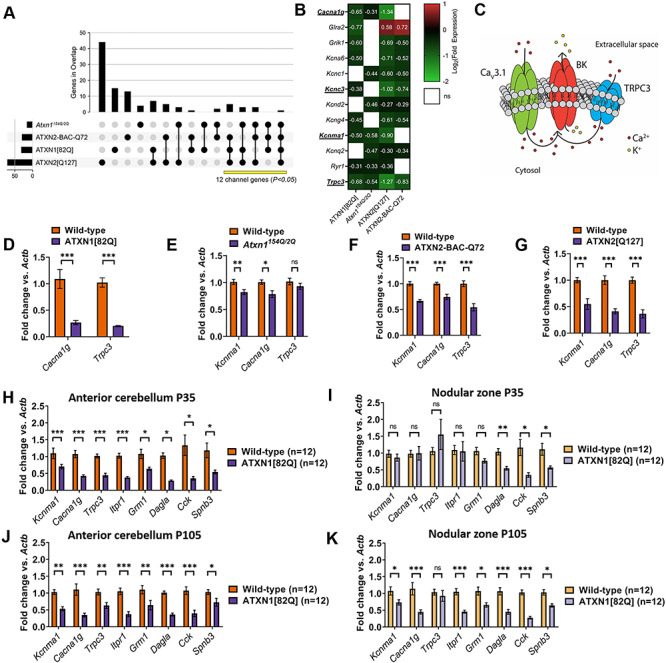
A functionally related module of channels is dysregulated uniquely in the anterior cerebellum of SCA1 mice. (**A**) IUPHAR-recognized ion channel genes that are dysregulated across models of SCA1 and SCA2. Vertical bars represent the number of ion channel genes unique to each overlap, and each overlap is delineated by black dots and linkages below the graph. The total number of ion channel genes differentially expressed in each model is shown as horizontal bars. The number of the channels that are found in at least three of four models (yellow bar) is statistically significant, with *P*-value reflecting the likelihood of an equivalent number of channels (or more) being dysregulated in these overlaps by chance (see Materials and Methods section). (**B**) Twelve ion channel transcripts are among the genes that are differentially expressed in the original analyses from three or four models of SCA1 and SCA2. Four of these channel transcripts (*Cacna1g*, *Kcnc3*, *Kcnma1* and *Trpc3*) are known ataxia genes and are underlined. (**C**) Proposed ion channel excitability module, highlighting a possible role for Ca_v_3.1 (encoded by *Cacna1g*) and TRPC3 (*Trpc3*) in providing Ca^2+^ for activation of BK channels (*Kcnma1*). (**D**–**G**) qRT-PCR for *Kcnma1*, *Cacna1g* and *Trpc3* transcripts from cerebella of 15-week ATXN1[82Q] mice (D), 14-week *Atxn1^154Q/2Q^* mice (E), 16-week ATXN2-BAC-Q72 mice (F) and 24-week ATXN2[Q127] mice (G). These are ages at which Purkinje cell loss is not present in these models. (**H**–**K**) Quantitative real-time PCR (qRT-PCR) was performed in macrodissected cerebella from ATXN1[82Q] mice and wild-type controls at P35 from (H) anterior cerebellum or (I) nodular zone and at P105 from (J) anterior cerebellum or (K) nodular zone. *Denotes *P* < 0.05; **denotes *P* < 0.01; ***denotes *P* < 0.001; ns denotes *P* > 0.05; two-tailed Student’s *t*-test with Holm-Sidak correction for multiple comparisons.

To identify those ion channel genes whose dysregulation was likely to reflect a biologically relevant disease pathway for Purkinje neurons, we compared previously published cerebellar whole-transcriptome datasets from models of spinocerebellar ataxias type 1 and 2, as Purkinje neurons are prominently affected in both conditions (6,15). Comparison of shared differentially expressed genes using RNA sequencing or microarray studies from ATXN1[82Q] (16), *Atxn1^154Q/2Q^* (17), ATXN2[Q127] and ATXN2-BAC-Q72 models (18) revealed 12 pore-forming (alpha subunit) ion channel genes that are differentially expressed in at least three of the four models (Fig. 2A and B, Supplementary Material, Fig. S2A). For 11 of the 12 genes, there is consistent downregulation across models. Additional RNA sequencing studies performed in the *Atxn1^154Q/2Q^* model (19,20) demonstrate the dysregulation of many of the same channels (Supplementary Material, Fig. S2B). Among these 12 channel genes, there are four genes for which mutations are known to cause human ataxia syndromes, namely *Kcnma1*, *Cacna1g*, *Trpc3* and *Kcnc3* (21–24). Patients with mutations in genes encoding any of these four channels can have cerebellar atrophy (21–23,25), and Purkinje neuron loss has been confirmed in autopsy samples from patients with *Cacna1g* mutations (22), suggesting that the dysregulation of any one of these channels individually may be sufficient to produce Purkinje neuron degeneration. The current study’s finding that these ataxia-linked channel genes are dysregulated in other forms of ataxia raises the possibility that the dysregulation of these channel genes could be a root cause for Purkinje neuron pathology in cerebellar ataxia.

**Figure 3:**
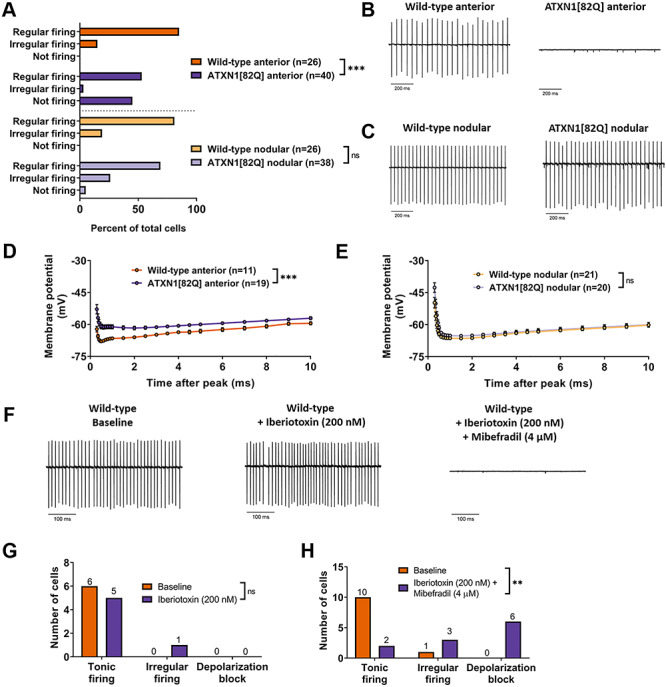
Regionally dysregulated ion channel genes form a functional module critical for Purkinje neuron pacemaking. (**A**) The distribution of regularly firing, irregularly firing and non-firing cells was recorded for Purkinje neurons in the anterior cerebellum and nodular zone. (**B**) Representative trace from a tonic firing wild-type and non-firing ATXN1[82Q] Purkinje neuron in the anterior cerebellum at P35. (**C**) Representative trace from wild-type and ATXN1[82Q] Purkinje neurons in the nodular zone at P35. (**D** and **E**) Membrane potential measurements during the AHP in wild-type Purkinje neurons (D) and ATXN1[82Q] Purkinje neurons (E) in the anterior cerebellum at P35. (**F**) Representative traces of wild-type Purkinje neurons in the anterior cerebellum at P35. Traces are shown at baseline (left), after perfusion of 200 nM iberiotoxin (middle) and after perfusion of 200 nM iberiotoxin +4 μM mibefradil. (**G** and **H**) Summary distribution of regularly firing, irregularly firing and non-firing Purkinje neurons before and after perfusion of 200 nM iberiotoxin (G) and after 200 nM iberiotoxin +4 μM mibefradil (H). **Denotes *P* < 0.01; ***denotes *P* < 0.001; ns denotes *P* > 0.05; Chi-square test (A, G and H); two-way repeated measures ANOVA with Holm-Sidak correction for multiple comparisons (D and E).

Among these four channel genes, the *Kcnma1*-encoded large-conductance calcium-activated K^+^ (BK) channel has emerged as a key player in multiple forms of spinocerebellar ataxia, namely SCA1, SCA2 and SCA7 (26–29). Notably, a recent study in a mouse model of SCA7 demonstrated that it is not only dysregulation of BK but also loss of Ca^2+^ homeostasis that promotes Purkinje neuron degeneration (29). This is consistent with the observation that the severe Purkinje neuron dysfunction observed in these SCA models cannot be recapitulated by targeted pharmacologic blockade of BK in wild-type neurons (30) but is readily apparent with intracellular Ca^2+^ chelation (31), which lowers intracellular free [Ca^2+^] needed for BK channel activation (32). Given that our analysis of SCA1 and SCA2 models reveals dysregulation of not just *Kcnma1*, but also the ataxia-linked Ca^2+^ channel genes *Cacna1g* and *Trpc3*, we chose to focus on these three channels and to expore the hypothesis that severe Purkinje neuron dysfunction observed in SCA1 might arise due to synergistic dysfunction of an ion channel module that contains BK and these Ca^2+^ sources (Fig. 2C). Transcript levels of these three genes (*Kcnma1*, *Cacna1g* and *Trpc3*) were directly assessed using quantitative reverse transcriptase (qRT)-PCR from the cerebella of ATXN1[82Q], *Atxn1^154Q/2Q^*, ATXN2-BAC-Q72 and ATXN2[Q127] models, and the reductions in transcripts were broadly consistent with the transcriptomic data (Fig. 2D–G), with reduction in *Kcnma1* from ATXN1[82Q] mice having been previously described (26).

To investigate whether coordinate dysregulation of this module may explain accelerated degeneration of Purkinje neurons from the anterior cerebellum, we performed qRT-PCR using macrodissected anterior cerebellum and nodular zone and investigated the expression of a panel of genes implicated in SCA1 pathogenesis. These genes included the ion channel module described above (*Kcnma1*, *Cacna1g* and *Trpc3*) as well as *Cck* (16), *Dagla* (33), *Spnb3*, *Itpr1* (34) and *Grm1* (35). At P35, when neurodegeneration was evident only in the anterior cerebellum, *Kcnma1*, *Cacna1g*, *Trpc3*, *Itpr1* and *Grm1* were downregulated only in the anterior cerebellum, while *Cck*, *Dagla* and *Spnb3* were downregulated in both regions (Fig. 2H and I). At P105, when neurodegeneration had also begun in the nodular zone, *Kcnma1*, *Cacna1g*, *Itrp1* and *Grm1* were also downregulated in the nodular zone (Fig. 2J and K). Taken together, these findings demonstrate tight spatiotemporal correlation between downregulation of an ion channel module and the onset of Purkinje neuron degenerations. They also suggest the critical genes in this module to be *Itpr1*, *Cacna1g* and *Kcnma1* and support a potential role for *Grm1* and *Trpc3*.

### Regionally dysregulated ion channel genes form a functional module critical for Purkinje neuron pacemaking

To determine whether regional downregulation of ion channel genes correlates with abnormal Purkinje neuron physiology, acute slice patch clamp recordings were performed in Purkinje neurons from the anterior cerebellum and nodular zone in ATXN1[82Q] and wild-type mice at P35. As previously reported, Purkinje neurons from ATXN1[82Q] mice are hyperexcitable and lack normal pacemaking at P35, with disrupted pacemaking arising from depolarization block (26,36). We confirmed these prior findings of depolarization block in up to 50% of Purkinje neurons in the anterior cerebellum. Surprisingly, there were no differences in firing between wild-type and ATXN1[82Q] Purkinje neurons in the nodular zone (Fig. 3A–C). Additional analysis of firing properties from all firing cells, namely frequency (Supplementary Material, Fig. S3A) and coefficient of variation (CV) (Supplementary Material, Fig. S3B), also revealed no difference in the nodular zone. A reduction in the after-hyperpolarization (AHP) has been suggested as the cause for depolarization block in ATXN1[82Q] Purkinje neurons (26,36), and there was a robust reduction in the AHP in ATXN1[82Q] Purkinje neurons from the anterior cerebellum (Fig. 3D) but not the nodular zone (Fig. 3E). Taken together, these findings demonstrate that there is Purkinje neuron hyperexcitability and dysfunction only in the anterior cerebellum at P35, consistent with the regionally restricted downregulation of *Kcnma1* and its potential Ca^2+^ sources.

Canonically, P/Q-type Ca^2+^ channels are thought of as the primary Ca^2+^ source that recruits calcium-activated K^+^ channels during the Purkinje neuron action potential (37). However, the proposed ion channel module does not include P/Q-type channels but does include other Ca^2+^ channels, namely the T-type Ca^2+^ channel Ca_V_3.1 (encoded by *Cacna1g*) and the type 1 inositol 1,4,5-triphosphate (IP3) receptor (encoded by *Itpr1*). Pharmacologic ion channel blockade was utilized in wild-type Purkinje neurons to determine whether these channels operate as upstream Ca^2+^ sources for BK in its role as a regulator of Purkinje neuron pacemaking. Consistent with prior studies (30,31), isolated blockade of BK with saturating concentrations of iberiotoxin was not sufficient to produce depolarization block (Fig. 3F and G). However, when BK channels were blocked along with T-type Ca^2+^ channels (using mibefradil at a dose specific for T-type channels in Purkinje neurons (38)), wild-type Purkinje neurons became irregular or underwent depolarization block (Fig. 3F and H). These data suggest that downregulation of BK along with one or more of these Ca^2+^ sources could be sufficient to explain the observed depolarization block, and also demonstrate that this group of ion channel genes comprises a functional module critical for Purkinje neuron pacemaking.

### Higher Cic expression in Purkinje neurons from the anterior cerebellum of SCA1 mice is associated with repression of key ion channel genes

**Figure 4:**
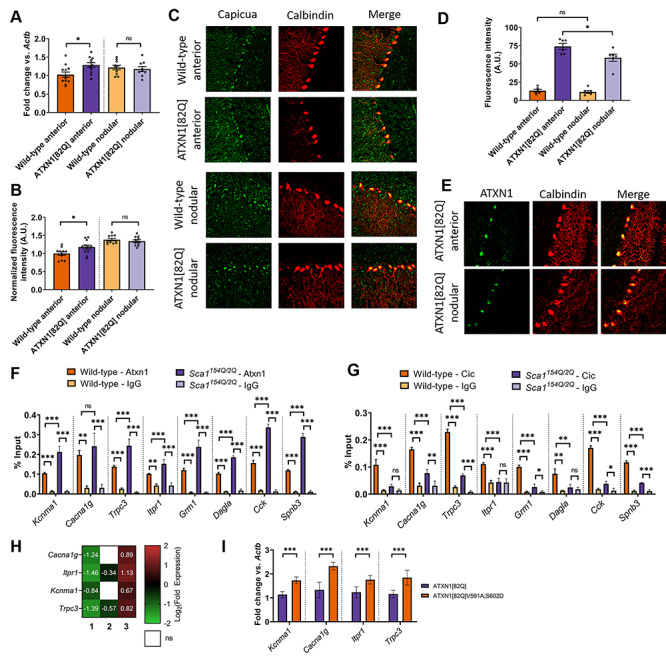
Higher Cic expression in Purkinje neurons from the anterior cerebellum of SCA1 mice is associated with the repression of key ion channel genes. (**A**) Quantification of relative expression of *Cic* mRNA in the anterior cerebellum and nodular zone of ATXN1[82Q] and wild-type cerebella at P35. (**B**) Quantification of relative expression of Cic protein in the somata of ATXN1[82Q] and wild-type Purkinje neurons at P35, normalized to values from wild-type anterior cerebellum. (**C**) Representative confocal images taken from ATXN1[82Q] and wild-type Purkinje neurons at P35 after immunostaining for Cic (green) and calbindin (red, to mark Purkinje neurons). (**D**) Quantification of relative expression of ATXN1 protein in the somata of ATXN1[82Q] and wild-type Purkinje neurons at P35. (**E**) Representative confocal images taken from ATXN1[82Q] mice at P35 after immunostaining for ATXN1 (green) and calbindin (red, to mark Purkinje neurons). (**F** and **G**) qChIP demonstrating the association of Cic and Atxn1 at the promoter of SCA1-associated genes from sonicated chromatin derived from P14 whole cerebellar extracts. Binding, represented as % input (*y*-axis) demonstrated for Atxn1 (F) and Cic (G) comparing their relative binding on SCA1-associated genes in *Atxn1^154Q/2Q^* mice and wild-type controls and binding over background relative to their respective isotype control IgG (rabbit IgG). (**H**) Log2 transformation of fold change expression demonstrating the role for the ATXN1-Cic complex in the dysregulation of *Cacna1g*, *Itpr1*, *Kcnma1* and *Trpc3*. Each column represents a distinct comparison (numbered by column): 1. ATXN1[82Q] relative to wild-type; 2. ATXN1[82Q]V591A;S602D relative to wild-type and 3. ATXN1[82Q]V591A;S602D relative to ATXN1[82Q]. (**I**) qRT-PCR for *Kcnma1*, *Cacna1g*, *Itpr1* and *Trpc3* from ATXN1[82Q]V591A;S602D and ATXN1[82Q] cerebella. *Denotes *P* < 0.05; **denotes *P* < 0.01; ***denotes *P* < 0.001; ns denotes *P* > 0.05; two-tailed Student’s *t*-test (A and C); two tailed-Student’s *t*-test with Holm-Sidak correction for multiple comparisons (D–I).

Having demonstrated the critical role for this ion channel module in Purkinje neurons, and knowing that its downregulation is tightly correlated with Purkinje neuron degeneration, we set out to explore the mechanism by which polyglutamine-expanded ATXN1 downregulates this ion channel module. We focused specifically on the transcriptional repressor Cic, which forms a native complex with ATXN1 that is central to SCA1 pathogenesis (39,40). Regional analysis of Cic mRNA (Fig. 4A) revealed that *Cic* expression is 26% higher in the anterior cerebellum at P35 (ATXN1[82Q]: 1.286 ± 0.067, wild-type: 1.026 ± 0.071). Previous reports have suggested that cerebellar Cic expression is largely restricted to immature granule cells (41), so the cell-type specificity of this effect was confirmed by immunohistochemistry, which showed an 18.0% higher nuclear Cic signal in anterior cerebellar Purkinje neurons from ATXN1[82Q] mice (Fig. 4B and C) P35 (ATXN1[82Q]: 1.180 ± 0.026, wild-type: 1.000 ± 0.047). Canonically, Cic function is regulated through control of Cic protein abundance, with steady-state levels set by binding to stabilizing partners. Cic and ATXN1 mutually stabilize each other, and an increase in Cic potein levels would therefore be expected to increase steady-state ATXN1 protein levels (39). We examined ATXN1 protein levels by immunohistochemistry in the anterior cerebellum and nodular zone in ATXN1[82Q] mice and wild-type mice and identified higher levels of ATXN1 in solely the anterior cerebellum of ATXN1[82Q] mice at P35 (Fig. 4D and E, Supplementary Material, Fig. S4A). These findings demonstrate that modestly greater Cic expression is associated with higher ATXN1 protein levels in the anterior cerebellum and correlates with dysregulated ion channel gene expression and Purkinje neuron degeneration in this region of the cerebellum in ATXN1[82Q] mice.

Because Cic functions principally as a transcriptional repressor, we tested the hypothesis that Cic might exert its effect on ion channel genes through increased promoter binding in the presence of polyglutamine-expanded ATXN1. We also hypothesized that this effect would be unique to SCA1-associated ion channel genes, given their unique downregulation in the anterior cerebellum (see Fig. 2D and E). Using the *Atxn1^154Q/2Q^* model of SCA1, where expression of Atxn1 is at endogenous levels (42), we found that Atxn1 showed robust polyglutamine length-dependent increases in baseline binding to all SCA1-associated genes, including both ion channel genes and non-channel genes (Fig. 4F, Supplementary Material, Fig. S4B and C). Additionally, we found that Cic binding for all genes was above IgG background but that there was actually a polyglutamine length-dependent decrease in promoter occupancy for all SCA1-associated genes (Fig. 4G, Supplementary Material, Fig. S4D and E). Taken together, these findings demonstrate that both SCA1-associated ion channel and non-channel SCA1 genes are Cic and Atxn1 targets but that polyglutamine-expanded Atxn1 shows increased promoter binding with a paradoxical reduction in Cic binding.

To test whether the dysregulation of ion channel genes is due to the actions of the ATXN1-Cic complex, we explored the expression of channel genes in the ATXN1[82Q]V591A;S602D mouse model (9). This mouse expresses polyglutamine-expanded ATXN1 with point mutations in the domain of ATXN1 that interacts with Cic. Comparison of expression levels for pore-forming (alpha subunit) ion channel genes from ATXN1[82Q], ATXN1[82Q]V591A;S602D and wild-type mice at 12 weeks of age revealed the dysregulation of many channel genes to be at least partially dependent on the ATXN1-Cic complex (Supplementary Material, Fig. S4F–I). Of note, there was statistical normalization of *Kcnma1* and *Cacna1g* expression as well as incomplete rescue of *Itpr1* and *Trpc3* in ATXN1[82Q]V591A;S602D mice (Fig. 4H). Analysis of expression levels for these four ion channel genes was confirmed by qPCR at P35, an early time point in disease in ATXN1[82Q] mice, revealing that there were higher cerebellar transcript levels of *Kcnma1*, *Cacna1g*, *Itpr1* and *Trpc3* in ATXN1[82Q]V591A;S602D cerebella relative to ATXN1[82Q] cerebella (Fig. 4I). These findings demonstrate that the Cic-ATXN1 interaction is crucial for ion channel gene dysregulation in ATXN1[82Q] mice.

Altogether, these data indicate that neither Cic nor Atxn1 show polyglutamine-dependent promoter occupancy effects that are unique to ion channel genes. This is in spite of a clear correlation between increased Cic expression in the anterior cerebellum and ion channel gene dysregulation, as well as the functional consequences of ATXN1-Cic interaction on ion channel gene expression. We therefore set out to explore whether changes in Cic expression are sufficient to explain anterior Purkinje neuron hyperexcitability and degeneration.

### Increased Cic expression results in Purkinje neuron hyperexcitability and accelerates Purkinje neuron degeneration

**Figure 5:**
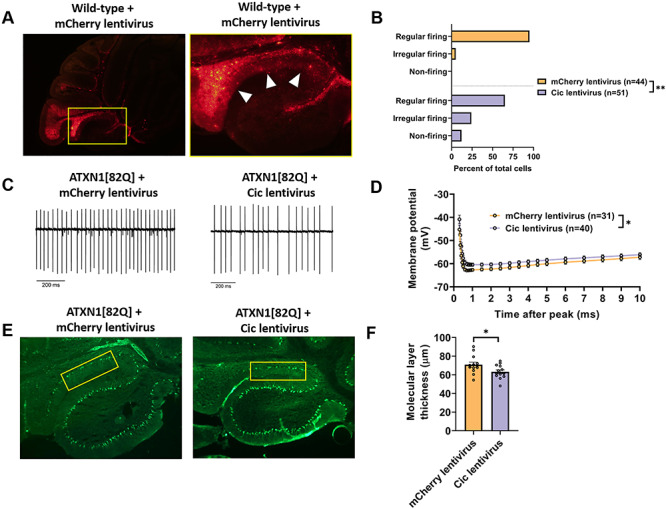
Increased Cic expression results in Purkinje neuron hyperexcitability and accelerates Purkinje neuron degeneration. (**A**) Stereotaxic injection of a lentiviral construct containing mCherry results in widespread expression in lobule IX, but not in lobule X, 10 days after injection. (**B**) The distribution of regularly firing, irregularly firing and non-firing Purkinje neurons in the nodular zone of ATXN1[82Q] mice at 10 days post-injection with Cic lentivirus or mCherry lentivirus. (**C**) Representative traces of ATXN1[82Q] Purkinje neuron spiking in the nodular zone at 10 days post-injection with Cic lentivirus or mCherry lentivirus. (**D**) Quantification of the AHP from ATXN1[82Q] Purkinje neurons in the nodular zone at 10 days post-injection with Cic lentivirus or mCherry lentivirus. (**E**) Representative images of the nodular zone of ATXN1[82Q] mice at 70 days post-injection with Cic lentivirus or mCherry lentivirus. Yellow box indicates analyzed area of lobule IX. (**F**) Quantification of molecular layer thickness measurements as illustrated in panel (E). *Denotes *P* < 0.05; **denotes *P* < 0.01; Chi square test (B); two-tailed Student’s *t*-test (F).

To investigate the causal connection between regional differences in Cic expression and ion channel dysregulation, we utilized a lentiviral vector to increase Cic expression in the nodular zone. When Cic lentivirus was co-injected with mCherry lentivirus, there was reliable overexpression of mCherry within the posterior half of lobule IX within the nodular zone (Fig. 5A), so patch clamp recordings were performed in Purkinje neurons from this area. At 10 days post-injection, a significant proportion of Purkinje neurons from lobule IX was found to display irregular spiking or depolarization block in ATXN1[82Q] mice injected with Cic lentivirus (Fig. 5B and C). Of note, lentiviral transduction by itself led to a greater degree of irregularity, as Purkinje neurons from an area that robustly expressed mCherry were more irregular than those in an area that was poorly transduced (Supplementary Material, Fig. S5A). Despite this, there was still a clear effect of higher Cic expression on the degree of irregularity (Supplementary Material, Fig. S5B). Cic-dependent disruptions in Purkinje neuron pacemaking occurred in association with a reduction in the AHP (Fig. 5D). These findings demonstrate that increasing Cic levels makes ATXN1[82Q] Purkinje neurons hyperexcitable.

Next, we investigated whether increased expression of Cic in the nodular zone impacted the kinetics of Purkinje neuron degeneration. Lentiviral Cic expression at 70 days post-injection was significantly attenuated as compared with 10 days post-injection (not shown). Nevertheless, in mice where the reporter gene mCherry was still clearly present at 70 days post-injection, Cic levels in Purkinje neuron nuclei showed modest but statistically insignificant greater level of Cic expression relative to mice injected with mCherry lentivirus alone (Supplementary Material, Fig. S5C). Given that this modest increase mirrored the modestly higher expression of Cic in the anterior cerebellum of ATXN1[82Q] mice (see Fig. 4), we aimed to assess its effect on Purkinje neuron degeneration in the nodular zone. Importantly, we found that ATXN1[82Q] mice injected with *Cic* lentivirus showed accelerated nodular zone Purkinje neuron degeneration at 70 days post-injection (Fig. 5E and F). These data demonstrate that only modestly greater expression of Cic and Cic-dependent hyperexcitability are likely to be responsible for accelerated degeneration of anterior cerebellar Purkinje neurons in ATXN1[82Q] mice.

## Discussion

Selective neuronal vulnerability is a universal feature of neurodegenerative disease, but the relevant pathways that differentiate affected cell types from protected cell types are not well understood. By examining sub-populations within a prominently affected cell type (Purkinje neurons), we identified a key disease pathway for the pathogenesis of SCA1. These findings not only provide important insights into the pathobiology of spinocerebellar ataxia but also validate the importance of cell subtype-level analysis as a platform for pathway discovery in neurodegenerative disease.

### Regional patterns of vulnerability in cerebellar degeneration

The current study not only confirms prior observations of an antero-posterior gradient of Purkinje neuron pathology in this model of SCA1 (10) but also adds to a robust literature exploring this phenomenon more widely in cerebellar ataxia syndromes. The current study did not explore regional differences in Cic expression or Purkinje neuron degeneration in other models of SCA1 or in SCA1 patients, and additional studies are required. Of note, ion channel gene dysregulation and hyperexcitability is observed in both anterior cerebellar and nodular zone Purkinje neurons from the *Atxn1^154Q/2Q^* model prior to the onset of neurodegeneration (43), suggesting that the findings from the ATXN1[82Q] model may not generalize to this model of SCA1. Regional differences in Purkinje neuron degeneration have not been explored in patients with SCA1, though a high-resolution MRI study in patients with SCA2 revealed a pattern of anterior-predominant degeneration that correlates with the degeneration of homologous murine structures in the current study (44).

Human imaging and autopsy studies have suggested that the nodular zone is more resistant to degeneration caused by both genetic and acquired insults (45,46). Studies in models of Niemann-Pick type C disease have suggested that this may stem from intrinsic differences in the resilience of Purkinje neurons across the antero-posterior axis, including differences in basal expression of genes that modulate protein homeostasis (47) or inflammation (48). Our findings support a different hypothesis for SCA1: rather than simply being more vulnerable to stress, anterior Purkinje neurons have a different regulatory program for key cellular functions (intrinsic excitability and its control by Cic in SCA1) that can be acted upon by relevant pathogenic proteins (polyglutamine-expanded ATXN1).

Several whole-transcriptome expression studies support the idea that tissue-specific gene expression networks can form a substrate for neurodegeneration, including in spinocerebellar ataxias broadly (13) and in this model of SCA1 specifically (16). In the current study, a more limited panel of genes is explored, but there is nevertheless a clear ‘module’ of ion channel genes that together mediate a specific cellular function and are coordinately regulated. Furthermore, the current study suggests that Cic is the regulator for this module, which not only establishes a novel role for Cic as a regulator of excitability in Purkinje neurons but also provides a mechanistic link between polyglutamine-expanded ATXN1 and abnormal Purkinje neuron physiology in SCA1.

Notably, the current study indicates that a relatively modest elevation of Cic (~20% in the anterior cerebellum) is sufficient to produce Purkinje neuron hyperexcitability and degeneration, but also indicates that there are regional differences in tolerance to Cic in ATXN1[82Q] mice. This conclusion is supported by the fact that in spite of higher baseline Cic expression in the nodular zone when compared with the anterior cerebellum, a virally mediated increase in Cic expression within the nodular zone of ATXN1[82Q] mice accelerates degeneration. The reason for tolerance for higher Cic in the nodular zone remains unclear. Further study is also warranted to identify the underlying mechanism behind greater Cic expression in the anterior cerebellum of ATXN1[82Q] mice at P35, particularly given the fact that the current study demonstrates a change in expression mediated by *Cic* mRNA levels, while canonical descriptions of Cic regulation center around the regulation of Cic protein abundance (39,49). Finally, study of Cic dysregulation is warranted in older ATXN1[82Q] mice, as ion channel gene dysregulation occurs at P105 in the nodular zone and could also be associated with greater expression of Cic.

Overall, the current study’s finding that modest elevations in Cic trigger Purkinje neuron hyperexcitabilty and degeneration raise the potential for translation to human therapeutics. The current study complements recent findings in human patients with heterozygous truncation mutation in CIC, which led to a neurobehavioral disorder that was likely developmental in origin (50). It is clear that neurons across a variety of brain regions are very sensitive to CIC gene dosing and that CIC levels are likely to be tightly regulated throughout the lifespan. Because this regulation appears to break down in SCA1, treatments that target regulators of CIC (or target CIC itself) to modestly lower expression may represent promizing candidates for symptomatic and neuroprotective treatment in patients with SCA1.

### Purkinje neuron excitability and Cic in SCA1 pathogenesis

Cic forms a native complex with ATXN1 that is essential for SCA1 pathogenesis (9,39,40). While significant progress has been made in understanding the ATXN1-Cic complex, including a validated partial crystal structure (51), much less is known about the mechanisms by which this complex drives Purkinje neuron degeneration. Our study demonstrates that the dysregulation of Purkinje neuron excitability is one such mechanism, and specifically highlights a functionally related group of channels (*Cacna1g*, *Itpr1* and *Kcnma1*) that we also show to be critical for Purkinje neuron pacemaking.

**Figure 6:**
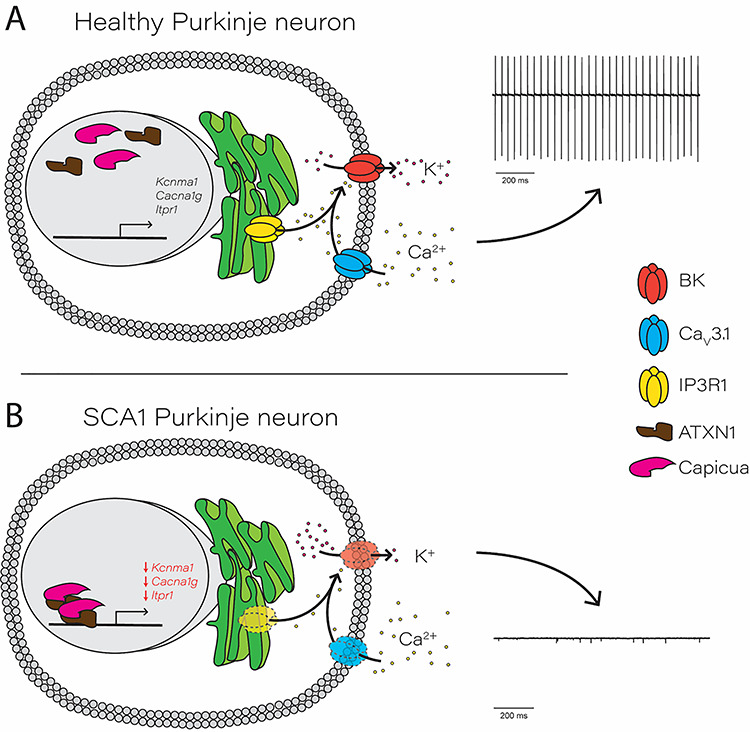
Proposed role for Capicua in transcriptional repression of an essential Purkinje neuron ion channel gene module. (**A**) In healthy, wild-type Purkinje neurons, Capicua (Cic) exerts basal transcriptional repression but allows transcription of key Purkinje neuron ion channel genes. Ca_v_3.1, located on the plasma membrane, and IP3R1, located on the endoplasmic reticulum membrane, act as Ca^2+^ sources for the BK channel. BK channel activation allows for normal potassium efflux during the action potential, which supports pacemaker firing (right). (**B**) In SCA1, increased repression of key Purkinje neuron ion channel genes is driven by the interaction between Capicua and polyglutamine-expanded ATXN1. Transcriptional repression results in reduced expression of ion channel proteins, leading to decreased BK channel activity and an inability to support pacemaker firing.

Abnormal Purkinje neuron excitability has emerged as an important pathogenic mechanism across multiple polyglutamine ataxias (12,14). Disrupted Purkinje neuron pacemaking in particular has been found in models of SCA1 (26,52), SCA2 (27,53), SCA3 (54), SCA6 (55) and SCA7 (29), with many studies demonstrating that treatments which improve pacemaking also slow Purkinje neuron degeneration. Purkinje neuron pacemaking depends on a suite of ion channels whose biophysical properties and expression levels are precisely tuned to produce a regenerative firing cycle (56). Our study supports two important conclusions about this suite of channels and their dysregulation in SCA1 (Fig. 6): (i) disrupted pacemaking in SCA1 occurs through downregulation of tightly coupled channels with a synergistic impact on spiking and (ii) the affected channels are under coordinate control through Cic.

In conclusion, our study identifies a likely Cic-dependent ion channel module whose critical role in Purkinje neuron pacemaking has not previously been described. The relevance of this module is likely to extend beyond SCA1, as our own analysis of whole-transcriptome data from SCA2 suggests that members of the module are dysregulated in SCA2, and a recent study in a model of SCA7 also demonstrates reduced transcript levels for all genes from this module (29). Given that ATXN2 and ATXN7 have not been described to act through Cic, it is tempting to speculate that the ion channel module we have identified represents an important shared pathway for Purkinje neuron pathology in many spinocerebellar ataxias. For this reason, our study in SCA1 highlights the potential for shared pathogenic processes with broad applicability to cerebellar disease.

## Materials and Methods

### Mice

All animal procedures were approved by the University of Michigan Committee on the Use and Care of Animals. ATXN1[82Q] transgenic mice (7) were maintained homozygous for the transgene on an FVB/NJ background (Jackson Labs). In all experiments involving ATXN1[82Q] transgenic mice, age- and sex-matched wild-type FVB/NJ mice were used as experimental controls. *Atxn1^154Q^* knock-in mice (42) were maintained on a C57Bl6J background (Jackson labs) by crossing *Atxn1^154Q/2Q^* males with *Atxn1^2Q/2Q^* wild-type females. In all experiments involving *Atxn1^154Q^* knock-in mice, *Atxn1^2Q/2Q^* littermates were used as wild-type experimental controls. ATXN2-BAC-Q72 mice were maintained on an FVB/NJ background (Jackson Labs). In all experiments involved ATXN2-BAC-Q72 mice, ATXN2-BAC-Q72 hemizygous mice were compared to ATXN2-BAC-Q72 null littermate controls. ATXN2[Q127] mice were maintained on a B6D2F1 hybrid background (Jackson labs). In all experiments involving ATXN2[Q127] mice, ATXN2[Q127] hemizygous mice were compared to ATXN2[Q127] null littermate controls.

### Electrophysiology

#### Solutions

Artificial CSF (aCSF) contained the following (in mM): 125 NaCl, 3.5 KCl, 26 NaHCO3, 1.25 NaH2PO4, 2 CaCl2, 1 MgCl2 and 10 glucose. For all recordings except for measurements of capacitance, pipettes were filled with internal recording solution containing the following (in mm): 119 K Gluconate, 2 Na gluconate, 6 NaCl, 2 MgCl2, 0.9 EGTA, 10 HEPES, 14 Tris-phosphocreatine, 4 MgATP, 0.3 tris-GTP, pH 7.3, osmolarity 290. For measurements of capacitance, pipettes were filled with internal recording solution containing the following (in mM): 140 CsCl, 2 MgCl2, 1 CaCl2, 10 EGTA, 10 HEPES, 4 Na2ATP, pH 7.3, osmolarity 287 mOsm.

#### Preparation of brain slices for electrophysiological recordings

Mice were anesthetized by isoflurane inhalation, decapitated, and the brains were submerged in pre-warmed (36°C) aCSF. Slices were prepared in aCSF held at 36°C on a VT1200 vibratome (Leica) while being continuously bubbled with carbogen (95% 02/5% CO_2_). Slices were prepared to a thickness of 300 μm. Once slices were obtained, they were incubated in continuously carbogen-bubbled aCSF for 45 min at 34°C. Slices were subsequently stored in continuously carbon-bubbled aCSF at room temperature until use.

#### Patch clamp recordings

Only slices from the cerebellar vermis were utilized for recordings, and slices were selected based on the ability to clearly visualize lobules II–X. Purkinje neurons from cerebellar lobules II–V or lobules IX–X were identified for patch clamp recordings using a 40x water-immersion objective and infrared differential interference contrast (IR-DIC) optics that were visualized using NIS Elements image analysis software (Nikon). Recordings were made 1–5 h after slice preparation in a recording chamber that was continuously perfused with carbogen-bubbled aCSF at 34°C at a flow rate of 2–3 mL/min. Borosillicate glass patch pipettes were pulled with a resistances of 3–5 MΩ. Recordings were performed on one of two patch clamp rigs, with equipment and technical specifications below.

- Rig 1: Data were acquired using an Axon CV-7B headstage amplifier, Axon Multiclamp 700B amplifier, Digidata 1440A interface and pClamp-10 software (MDS Analytical Technologies). All voltage data were acquired in the current-clamp mode with bridge balance compensation and filtered at 2 kHz.
- Rig 2: Data were acquired using an Axon CV-7B headstage amplifier, Axopatch 200B amplifier, Digidata 1440A interface and p-Clamp-10 software (MDS Analytical Technologies). All voltage data were acquired in the fast current-clamp mode (57) and filtered at 2 kHz.

For a given experiment, all data were acquired on the same rig to avoid experimental confounds arising from data acquisition. In all cases, acquired data were digitized at 100 kHz. Cells were rejected if the series resistance rose above 15 MΩ, with the majority of recordings having a series resistance of <10 MΩ. Cells were also rejected if the series resistance changed by >20% during recording. Voltage in this study is corrected for a liquid junction potential of +10 mV (58).

#### Analysis of cell capacitance

Determination of Purkinje neuron membrane capacitance was performed using a well-established method for analysis of a two-compartment equivalent circuit representing a Purkinje neuron (59) and has been described previously (28,29). Briefly, recordings in the anterior cerebellum and nodular zone were performed in the presence of 50 μM picrotoxin (cat. no. P1675, Sigma-Aldrich), and capacitative transients were obtained in voltage clamp mode using 1 s steps to −90 mV from a holding potential of −80 mV. Recordings were discarded if the measured input resistance was <100 MΩ. Voltage steps were performed 10 times and the recorded currents were averaged and low-pass filtered at 5 kHz. The decay of the capacitative transient was fit using a two-exponential decay function described as follows (Eq. 1):

(1) \begin{equation*} I(t)={A}_1{e}^{-\frac{t}{\tau_1}}+{A}_2{e}^{-\frac{t}{\tau_2}}. \end{equation*}

The constants from each cell’s decay function were then used to obtain four parameters: *C*_1_ (representing the capacitance of the soma and main proximal dendrites, Eq. 2), *C*_2_ (representing the capacitance of the distal dendritic arbor, Eq. 3), *R*_1_ (representing the pipette access resistance and internal resistance of the soma and proximal dendrite, Eq. 4) and *R*_2_ (representing the composite internal resistance of dendritic segments separating the distal dendritic arbor from the main proximal dendritic segments, Eq. 5). This was done as follows [equations described previously (59)]:

(2) \begin{equation*} {C}_1=\frac{\tau_1{\left({A}_1+{A}_2\right)}^2}{A_1\Delta V} \end{equation*}

(3) \begin{equation*} {C}_2=\frac{A_2{\tau}_2}{\Delta V} \end{equation*}

(4) \begin{equation*} {R}_1=\frac{\Delta V}{A_1+{A}_2} \end{equation*}

(5) \begin{equation*} {R}_2=\frac{\Delta V}{A_2}-\frac{\Delta V}{A_1+{A}_2}. \end{equation*}

In our measurements of cell capacitance, |*C*_1_ + *C*_2_| is reported in Figure 1B and C.

Of note, modeling studies have suggested that the reduced two-compartment Purkinje neuron model is statistically inadequate for estimating membrane capacitance in mature Purkinje neurons (60). To ensure that our estimates based on the two-compartment model were internally consistent, these estimates were compared with capacitance estimates utilizing a different method wherein the capacitance is estimated from the area underneath the capacitative transient (26). Comparison of total cell capacitance by both methods revealed that there was no statistically significant difference in estimated cell capacitance based on the method used, suggesting that the two compartment model could reliably estimate Purkinje neuron capacitance in our recordings (Supplementary Material, Fig. S1C–F).

#### Analysis of firing properties

Electrophysiology data were analyzed offline using Clampfit 10.2 software (Molecular Devices). Firing frequency and CV calculations were performed using a 10 s recording obtained in the cell-attached configuration ~5 min after formation of a stable seal. The CV was calculated as follows:

$$ \mathrm{CV}=\frac{\mathrm{Standard}\ \mathrm{Deviation}\ \mathrm{of}\ \mathrm{Interspike}\ \mathrm{Interval}}{\mathrm{Mean}\ \mathrm{Interspike}\ \mathrm{Interval}}. $$

For Figures 3A, G and H and 5B, cells were classified as either regular firing, irregular firing or depolarization block/non-firing. For these figures, irregular firing cells were defined as those cells whose CV was greater than the mean + 1 standard deviation CV of Purkinje neurons from the corresponding experimental control. Depolarization block/non-firing cells were defined as cells that were not firing in the cell-attached configuration but could fire a train of >5 action potentials in response to current injection in the whole-cell configuration. Cells which were not firing in the cell-attached configuration and were unable to fire a train of >5 action potentials in the whole-cell configuration were discarded.

#### AHP analysis

Analysis of the AHP was performed on recordings where the cell was held at −80 mV and injected with a series of escalating 1 s current pulses. The AHP was analyzed using the first spike from the first trace where there was no greater than a 50 ms delay to the spike from current injection onset. Reported AHP values reflect the membrane potential at specified time intervals after the peak of the spike.

#### Pharmacology

In some electrophysiology recordings, mibefradil dihydrochloride hydrate (cat. no. M5441, Sigma Aldrich) was used at a concentration of 4 μM to fully inhibit Ca_V_3 family T-type Ca^2+^ channels (38), and iberiotoxin (cat. no. STI-400, Alomone Labs) was used at 200 nM to fully inhibit BK channels.

### Tissue immunohistochemistry

Mice were anesthetized with isoflurane and brains were removed, fixed in 1% paraformaldehyde for 1 h, immersed in 30% sucrose in PBS and sectioned on a CM1850 cryostat (Leica). Parasagittal sections of 14 μm were processed for immunohistochemistry as described previously (26). Sections were kept stored at −80°C prior to antibody staining and imaging.

For molecular layer thickness measurements, Purkinje neurons were labeled with mouse anti-calbindin (1:1000, cat. no. C9848, Sigma-Aldrich) and goat anti-mouse Alexa488 conjugated secondary antibody (1:200, ref. no. A11001, Life Technologies Invitrogen). Sections were imaged using an Axioskop 2 plus microscope (Zeiss) at either 10x or 20x magnification. Measurements were performed using cellSens Standard image analysis software (Olympus). To measure molecular layer thickness in the anterior cerebellum, a line was drawn that measured 100 μm from the depth of the primary fissure along the length of the fissure, and the distance between the end of this line and the nearest Purkinje neuron cell body in lobule V was reported as the molecular layer thickness. To measure molecular layer thickness in the nodular zone, a line was drawn that measured 50 μm from the depth of the lobule IX/X fissure, and the distance between the end of this line and the nearest Purkinje neuron cell body in lobule IX or X was reported as the molecular layer thickness. Measurements of molecular layer thickness were performed in two sections per animal, and the reported molecular layer thickness for each animal is the mean of these two measurements. Sample preparation, imaging and measurement for molecular layer thickness were performed with experimenter blind to genotype and treatment.

For double immunofluorescence experiments, Cic was labeled with rabbit anti-Capicua/CIC antibody (1:1000, cat. no. ab123822, Abcam) and goat anti-rabbit AlexaFluor 488 conjugated secondary antibody (1:200, cat. no. A11008, Life Technologies Invitrogen), while Purkinje cells were labeled with mouse anti-Calbindin antibody (1:1000, cat. no. C9848, Sigma-Aldrich) and goat anti-mouse AlexaFluor 594 (1:200, cat. no. A11005, Life Technologies Invitrogen). To quantify the intensity of Cic, images were analyzed using Plot Profile in ImageJ. To do this, a line was drawn across the Cic-positive area in the soma and the area under the curve was measured, and the somatic Cic intensity for that cell was defined as the area under the curve divided by the length of the line. Somatic Cic intensity was measured in ~ 6 cells from both the anterior or nodular zone for each animal, and the mean of these values from the anterior cerebellum and nodular zone was determined for each animal. Those mean intensities were then all normalized to the average Cic intensity values from the anterior cerebellum of all wild-type animals to produce the values reported in Figure 4C. Sample preparation, imaging and analysis were performed with experimenter blind to genotype.

### Transcriptome-level analysis of ion channel gene expression

#### RNA sequencing for SCA2 mice

Cerebella from 6-week-old ATXN2[Q127] and wild-type littermates (16 animals in each group) or 8-week-old ATXN2-BAC-Q72 and wild-type littermates (4 animals in each group) were used for RNA sequence analyses. Total RNA was isolated using miRNeasy Mini Kit (Qiagen Inc., USA) according to the manufacturer’s protocol. RNA quality was determined using the Bioanalyzer 2100 Pico Chip (Agilent). Samples with an RNA integrity number (RIN) >8 were used for library preparation using Illumina TrueSeq Stranded Total RNA Sample Prep with Ribo-Zero rRNA Removal Kit for mouse. Single-end 50-bp reads were generated on a Hiseq 2000 sequencing machine at the University of Utah Microarray and Genomic Analysis Shared Resource using Illumina Version 4 flow cells. Reads were then aligned to the mouse reference genome (mm10) by Novoalign (http://www.novocraft.com), and differentially expressed genes were identified as previously described (18).

#### Analysis of ion channel gene expression from SCA1 and SCA2 mice

Published data tables from a previous RNA sequencing analysis of gene expression in ATXN1[82Q] mice (16) and from a microarray analysis of gene expression in *Atxn1^154Q/2Q^* mice (17) were downloaded from the NCBI Gene Expression Omnibus (accession number GSE75778 and GSE991, respectively). Data tables for ATXN2[Q127] mice and ATXN2-BAC-Q72 mice were generated as described above. From these data tables, genes that were found to differentially expressed in the original analysis for each dataset were selected for further analysis, along with corresponding fold change expression and log_2_ conversion of the fold change expression. In Figure 2B, the log_2_ conversion of the fold change expression relative to each model’s respective wild-type control is plotted, and the log_2_ conversion of the fold change expression labeled for comparisons showing differential expression (rounded to two decimal places for visual clarity).

Data tables of differentially expressed genes were analyzed to identify shared transcripts across models using a custom script, which was executed using the Jupyter Notebook web application and running the Anaconda distribution of Python 3.6.3. This script and input data tables required to perform the analysis are available at https://github.com/chopravi/DiffEx_SCA1_SCA2. For each model, dysregulated channel genes were identified from the full list of differentially expressed genes in that model by searching the differentially expressed genes against the 270 genes found in mice from the IUPHAR classification for pore-forming (alpha subunit) ion channels (61). The lists of dysregulated channel genes in each model were compared to identify all possible overlaps between models, and these overlaps are represented in Figure 2A using an UpSet plot (62) along with an unscaled Venn diagram in Supplementary Material, Figure S2A. For Figure 2A, broken axes were created in Illustrator (Adobe) using the direct output of the custom script referenced above.

To determine whether there was a statistically significant number of dysregulated channel genes shared across models, we tested the null hypothesis that the number of channel genes shared between these models could occur by chance, given the number of channel genes that are differentially expressed in each model. Specifically, to evaluate the statistical significance of the channels dysregulated in any 3 models, we tested the null hypothesis that 12 or more ion channel genes could be obtained from the union of the overlaps between any 3 models by chance. The probability for this overlap was determined by performing 1000 simulations in which the appropriate number of channels was randomly sampled for each model from the IUPHAR database of all ion channels found in mice, at which point the number of shared channels in appropriate overlaps was counted. The null hypothesis was rejected and the number of channel genes that are dysregulated was deemed to be statistically significant if *P* < 0.05.

#### Analysis of RNA sequencing data from Atxn1^154Q/2Q^ mice

Published data tables from two previous RNA sequencing analyses of gene expression in *Atxn1^154Q/2Q^* mice were utilized. One was a study published by Driessen and colleagues (19), and data tables for cerebellum from 12-week-old *Atxn1^154Q/2Q^* mice were downloaded from the NCBI Gene Expression Omnibus (accession number GSE122099) and differentially expressed genes were selected based on the authors’ definition. The other was a study published by Friedrich and colleagues (20), from which data tables comparing 18-week-old vehicle-treated wild-type and *Atxn1^154Q/2Q^* mice were provided by the authors that differentially expressed genes were selected using an adjusted *P* < 0.01. From these data tables of differentially expressed genes, channel genes that were dysregulated in either study along with corresponding fold change expression and log_2_ conversion of the fold change expression were identified using the custom Python script described above. Log_2_ conversion of fold expression changes is presented for these dysregulated channel genes in Supplementary Material, Figure S2B.

#### Analysis of ion channel gene expression from ATXN1[82Q]V591A;S602D mice

RNA sequencing was performed and read counts were obtained from hemizygous ATXN1[82Q] mice, hemizygous ATXN1[82Q]V591A;S602D mice and wild-type controls as described previously (9). The read counts from this previously published dataset were used to perform differential gene expression analyses for the 270 IUPHAR pore-forming (alpha subunit) ion channel genes using the DESeq2 package (63) (version 1.24.0) in the R environment (version 3.6.1) with default parameters. From this analysis, dysregulated channel genes from ATXN1[82Q] mice were selected using an adjusted *P* < 0.01, along with corresponding fold change expression and log_2_ conversion of the fold change expression, using the custom Python script described above.

In Figure 4H, the log_2_ conversion of the fold change expression are presented for *Cacna1g*, *Itpr1*, *Kcnma1* and *Trpc3*. In Supplementary Material, Figure S4F–I, the log_2_ conversion of the fold change expression for all dysregulated channel genes from ATXN1[82Q] mice is presented. For both figures, three comparisons are plotted: (i) ATXN1[82Q] relative to wild-type FVB/NJ; (ii) ATXN1[82Q]V591A;S602D relative to wild-type FVB/NJ and (iii) ATXN1[82Q]V591A;S602D relative to ATXN1[82Q]. For Supplementary Material, Figure S4F–I, channel genes were subclassified using the custom Python script described above into those that are ATXN1-Cic complex dependent, ATXN1-Cic complex partially-dependent and ATXN1-Cic complex independent, defined as below.

- ATXN1-Cic complex-dependent channel genes: Channel genes whose dysregulation was abolished by disrupting ATXN1-Cic association are labeled as ATXN1-Cic complex-dependent channel genes. These genes met 2 criteria. First, they were differentially expressed in the ATXN1[82Q]V591A;S602D versus ATXN1[82Q] comparison. Second, they were not differentially expressed in the ATXN1[82Q]V591A;S602D versus wild-type FVB/NJ comparison. For Supplementary Material, Figure S4F and G, ATXN1-Cic complex-dependent channel genes are further subdivided into those that are downregulated in ATXN1[82Q] mice relative to wild-type and those that are upregulated in ATXN1[82Q] relative to wild-type
- ATXN1-Cic complex partially dependent channel genes: Channel genes whose dysregulation was partially rescued by disrupting ATXN1-Cic association are labeled as ATXN1-Cic complex partially dependent channel genes. These genes met 2 criteria. First, they were differentially expressed in the ATXN1[82Q]V591A;S602D versus ATXN1[82Q] comparison. Second, they were differentially expressed in the ATXN1[82Q]V591A;S602D versus wild-type FVB/NJ comparison.
- ATXN1-Cic complex independent channel genes: Channel genes whose dysregulation was not affected by disrupting ATXN/1Cic association are labeled ATXN1-Cic complex-independent channel genes. These channel genes were those which were not differentially expressed in the ATXN1[82Q]V591A;S602D versus ATXN1[82Q] comparison.

In both figures, the log_2_ conversion of the fold change expression is labeled for statistically significant comparisons (rounded to two decimal places for visual clarity).

### Gene expression by quantitative real time PCR (qRT-PCR)

#### Tissue preparation

For analysis of anterior cerebellum and nodular zone gene expression, mice were euthanized following anesthesia with isoflurane, and cerebella were removed and placed in ice-cold PBS. The cerebellar hemispheres were removed with a razor blade, and the vermis was then laid on its lateral aspect and the lobules were visualized under a dissecting scope. The anterior cerebellum and nodular zone were separated from the remaining cerebellar tissue using glass microelectrodes pulled for patch clamp recordings. Once tissue was isolated, it was flash-frozen in liquid nitrogen and stored at −80°C until the time of processing.

For analysis of whole cerebellar gene expression, mice were euthanized following anesthesia with isoflurane, and cerebella were removed and flash-frozen in liquid nitrogen. Tissue was stored at −80°C until the time of processing.

#### RNA Extraction, cDNA synthesis and quantitative real-time PCR

Total RNA from each harvested portion of mouse cerebellum or whole cerebellum was extracted using Trizol Reagent (Invitrogen) and subsequently purified using the RNeasy Mini Kit (Qiagen Inc., USA) following the manufacturer’s instructions. cDNA was synthesized from 1 μg of purified RNA using the iScript cDNA synthesis kit (Cat. no. 1708891, Bio-Rad). Quantitative real-time PCR assays were performed using the iQ SYBR Green Supermix (Cat. no. 1708880, Bio-Rad) in a MyiQ Single Color Real-Time PCR Detection System (Bio-Rad), with each reaction performed at a 20 μL sample volume in an iCycler iQ PCR 96-well Plate (Bio-Rad) sealed with Microseal optical sealing tape (Bio-Rad). The primers (5′-3′) used for qRT-PCR are listed below.

*Kcnma1* Forward: AGCCAACGATAAGCTGTGGT.

*Kcnma1* Reverse: AATCTCAAGCCAAGCCAACT.

*Cacna1g* Forward: GTCGCTTTGGGTATCTTTGG.

*Cacna1g* Reverse: TACTCCAGCATCCCAGCAAT.

*Trpc3* Forward: GAGGTGAATGAAGGTGAACTGA.

*Trpc3* Reverse: CGTCGCTTGGCTCTTATCTT.

*Itpr1* Forward: GGCAGAGATGATCAGGGAAA.

*Itpr1* Reverse: AGCTCGTTCTGTTCCCCTTC.

*Grm1* Forward: CCACCTCTGATGTAGTGCG.

*Grm1* Reverse: TGACACAGACTTGCCGTTAG.

*Dagla* Forward: CACCTTCGTCAAGCTGAGAG.

*Dagla* Reverse: AGAGGAACACTTTTAGACGGC.

*Cck* Forward: ACTGCTAGCGCGATACATC.

*Cck* Reverse: GGTCACTTATTCTATGGCTGGG.

*Spnb3* Forward: AGAACTGGGTTGCACTGTG.

*Spnb3* Reverse: CTTCCTTTTCCGCTCATTTTCC.

*Cic* Forward: CTTTCCCTAGCGCCACAG.

*Cic* Reverse: TACCCGATTCAAAAGACCCC.

Human ATXN1 Forward: CCAGCACCGTAGAGAGGATT.

Human ATXN1 Reverse: AGCCCTGTCCAAACACAAAA.

*Actb* Forward: CGGTTCCGATGCCCTGAGGCTCTT.

*Actb* Reverse: CGTCACACTTCATGATGGAATTGA.

The relative amount of transcript mRNA was determined using the comparative *C*_t_ method for quantitation for ATXN1[82Q] and *Atxn1^154Q/2Q^* mice (64) and standard curve quantitation for ATXN2[Q127] and ATXN2-BAC-Q72 mice (18) with *Actb* mRNA serving as the reference gene. *C*_t_ values for each sample were obtained in triplicate and averaged for statistical comparisons. Relative fold change data compared with *Actb* for Figure 2H–K are normalized to the respective macro-dissected cerebellar portion from wild-type mice. Otherwise, relative fold change data compared with *Actb* are normalized to whole cerebellum from wild-type mice.

### Fast chromatin immunoprecipitation

Quantitative chromatin immunoprecipitation (qCHIP) was performed as previously described (65) with minor modifications. A pool of isolated cerebellum tissue from P14 mice (three to six mice per genotype) was digested for 20 min with Pappain (SKU no. PAP, Brainbits) + DNase I (10 μg/ml; Sigma) followed by 10-fold dilution in PBS supplemented with 1% fetal bovine serum (FBS). After centrifugation (300 g, 5 min), the cell pellet was washed three times in PBS + 1% FBS and the resultant final pellet (containing a single cell suspension) was used to isolate chromatin. This suspension was sonicated until chromatin was sheared to a length of 200–500 bp. A quantity of sheared chromatin corresponding to one cerebellum from the chromatin pool was incubated with 50 μL of Rabbit anti-Atxn1 (kindly provided by Harry Orr), 2.5 μg Rabbit anti-Cap (cat. no. ab123822, Abcam) or 2.5 μg normalized Rabbit IgG (Santacruz) using Dynabeads (Invitrogen). After washing, elution and cross-link reversal, DNA from each ChIP sample and the corresponding input sample was purified.

Quantitative PCR (qPCR) was used to analyze the percentage of DNA recovered after ChIP from each sample. Each ChIP sample and a range of dilutions of the corresponding input sample (0.01–2% input) were quantitatively analyzed with gene-specific primers using the 7500 detection system (ABI) and SYBR qPCR Premix (Clontech). For each panel included in Figure 4 or Supplementary Material, Figure S4, the data presented are from an individual tissue preparation and immunoprecipitation. The primers (5′-3′) used for qPCR are listed below.

*Kcnma1* Forward: CATGGTCACCGGTATGATGA.

*Kcnma1* Reverse: ATATCCACGCGAACCATCTC.

*Cacna1g* Forward: TCACTTTGTTCCGGCTTCTT.

*Cacna1g* Reverse: TGCTGACCCCTTAGATCCTG.

*Trpc3* Forward: GCTGTGCAGGAATCAGACAA.

*Trpc3* Reverse: CATGAGGGTTCTGGGTCTCT.

*Itpr1* Forward: CCCAAACGTCCTCGACTCTA.

*Itpr1* Reverse: CACTCCAACCGTTTCCAACT.

*Grm1* Forward: TGCAGCTTTATTCCCCAATC.

*Grm1* Reverse: TCACCTTGAGAAAGGGATCG.

*Dagla* Forward: AGAACCATGCCAACCAACAT.

*Dagla* Reverse: GCGAGGGTGTCAGAAGACTC.

*Cck* Forward: GCTCCGAACAGCAGAGAAAC.

*Cck* Reverse: TCCCTCCCTTGACAGCTAGA.

*Spnb3* Forward: GTGATGACGGTTCCGAGAAT.

*Spnb3* Reverse: CCCTTTCTCTCGTCCTCCTT.

### Lentiviral transduction of Purkinje neurons

#### Viral vectors

Viral vectors were generated by and purchased from the University of Minnesota Viral Vector and Cloning Core (Minneapolis, MN). cDNA encoding either Capicua (Cic) or mCherry was packaged into a lentiviral vector under control of the human synapsin (hSyn) promoter. Myc-CICf was a gift from Huda Zoghbi (Addgene plasmid #48185; http://n2t.net/addgene:48185; RRID:Addgene_48 185) (51). Lentiviral titers were as follows: hSyn.Cic, 3.34 × 10^7^ viral particles/mL; hSyn.mCherry, 4.67 × 10^7^ viral particles/mL. hSyn.mCherry lentivirus was diluted to 3.33 × 10^7^ viral particles/mL in sterile phosphate buffered saline to facilitate equal delivery of volume and viral load into the cerebellar cortex.

#### Cerebellar delivery of lentivirus

Lentiviral vectors were delivered to the cerebellar cortex using stereotaxic surgical techniques. ATXN1[82Q] or wild-type controls at P28-P38 were used for surgery. Mice were anesthetized under 5% isoflurane inhalation and were maintained at 1.5% isoflurane for the duration of the procedure. A single craniotomy above the nodular zone was performed with a small drill burr at bregma −7.0 mm in the anterior/posterior plane. About 3.0 μL lentivirus was loaded into a 10 μL Hamilton syringe (BD Biosciences, San Jose, CA), which was then slowly lowered into the nodular zone to a depth of bregma −3.8 mm in the dorsal/ventral plane. A series of pilot injections using 3.0 μL of hSyn.mCherry lentivirus were used to confirm the surgical coordinates. For electrophysiological recordings and molecular layer thickness measurements, mice were injected with either 3.5 μL hSyn.mCherry (control) or a combination of 3.0 μL hSyn.Cic + 0.5 μL hSyn.mCherry for the verification of infection in lobule IX. Lentivirus was delivered at a rate of 500 nL/min using an injection pump (UMC4, World Precision Instruments, Inc., Sarasota, FL). After completion of delivery, the syringe was allowed to sit for 2 min before slight retraction, after which the syringe was left in place for six additional minutes to prevent backflow of the lentivirus into the syringe tract. After removal of the syringe, the scalp was sutured, and the mouse was monitored during recovery from anesthesia. Post-operative care was provided for at least 7 days after surgery, and carprofen (2.5 mg/kg subcutaneous) was administered for the first 72 h after surgery.

### Chemicals

Reagents and chemicals were obtained from Sigma-Aldrich unless otherwise specified.

### Statistical analysis

Statistical tests are described in the figure legends for all data. Data are expressed as mean ± SEM unless otherwise specified. Sample size is included in each figure panel, either by plotting of individual data points or by including the number of cells (*n*) within the figure panel. Studies were powered and analysis was performed assuming unequal variance between groups. Data were analyzed using GraphPad Prism (GraphPad) and Excel (Microsoft).

## Supplementary Material

Chopra_et_al_Supplementary_Figures_ddaa212
