# Supplementary material for: Altered Capicua expression drives regional Purkinje neuron vulnerability through ion channel gene dysregulation in spinocerebellar ataxia type 1
Source: Hum Mol Genet. 2020 Sep 23;29(19):3249–65. doi: 10.1093/hmg/ddaa212 (PMC7689299; doi:10.1093/hmg/ddaa212)
Supplement: Chopra_et_al_Supplementary_Figures_ddaa212 [file chopra_et_al_supplementary_figures_ddaa212.docx]

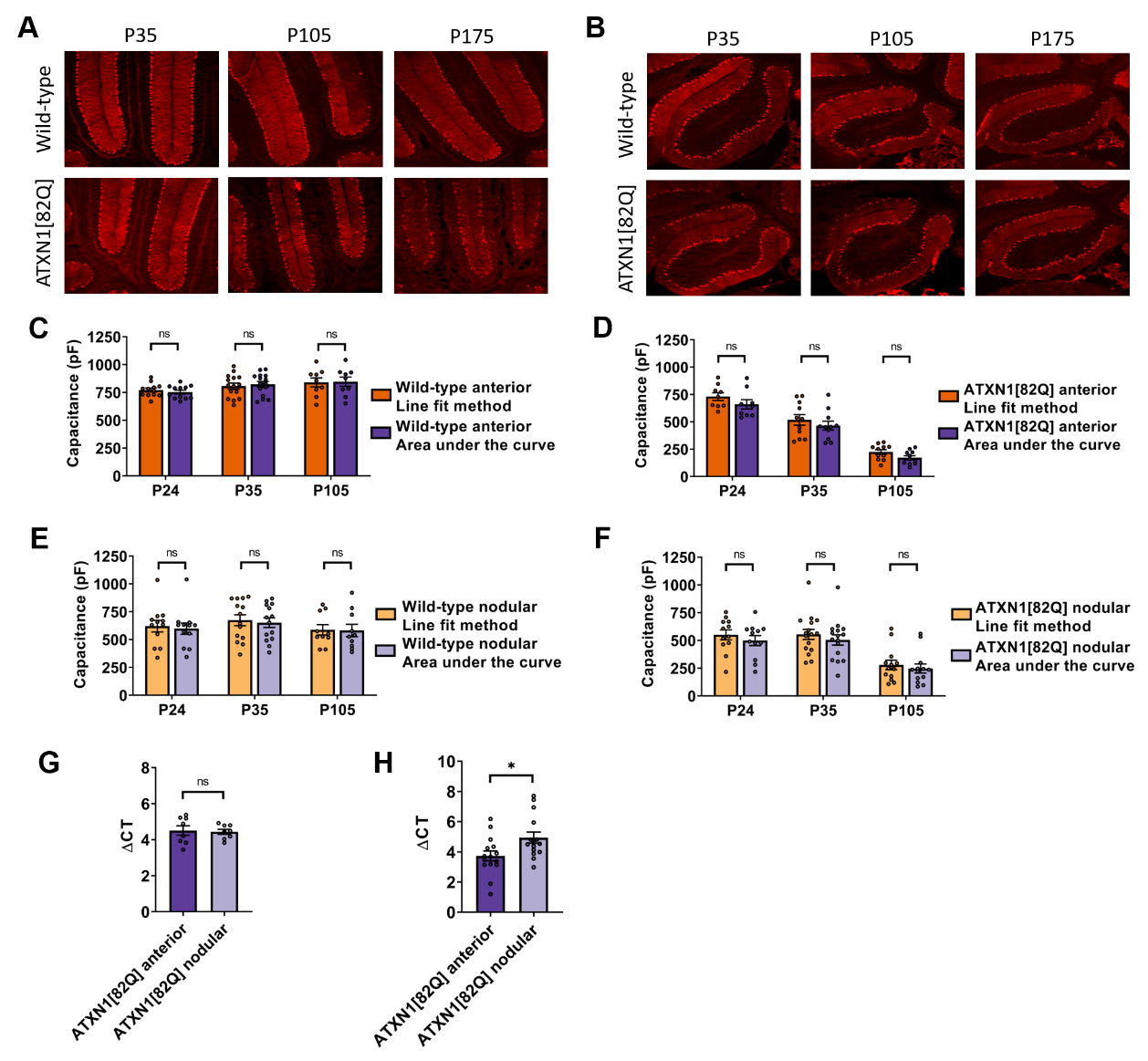
**Supplementary Figure 1. Detection of Purkinje neuron capacitance and cerebellar ATXN1[82Q] transgene expression**

(A) Representative images of the primary fissure (defining the posterior border of the anterior cerebellum). Anterior cerebellum molecular layer thickness was measured in the posterior aspect of lobule V. (B) Representive image of the nodular zone. Nodular zone molecular layer thickness was measured and the anterior aspect of lobule X. (C-F) Capacitance data in Figure 1B-1C was fit using a two-exponential decay function based on a model for the Purkinje neuron as a two-compartment equivalent circuit (59). Capacitance measurements from anterior cerebellum wild-type Purkinje neurons (C), anterior cerebellum ATXN1[82Q] Purkinje neurons (D), nodular zone wild-type Purkinje neurons (E), and nodular zone ATXN1[82Q] Purkinje neurons (F) were compared using the two-exponential decay function and an alternative method of measuring capacitance wherein area under the curve is taken from the current trace measured during a -10 mV voltage step from -80 mV to -90 mV. (G) Relative human ATXN1 transgene expression is displayed for macrodissected anterior cerebellum and nodular zone from ATXN1[82Q] mice at P35. (H) Relative human ATXN1 transgene expression is displayed for macrodissected anterior cerebellum and nodular zone from ATXN1[82Q] mice at P105. * denotes p<0.05; ns denotes p>0.05; two-way repeated measures ANOVA with Holm-Sidak correction for multiple comparisons (B-E); two-tailed Student’s t-test (F-G).


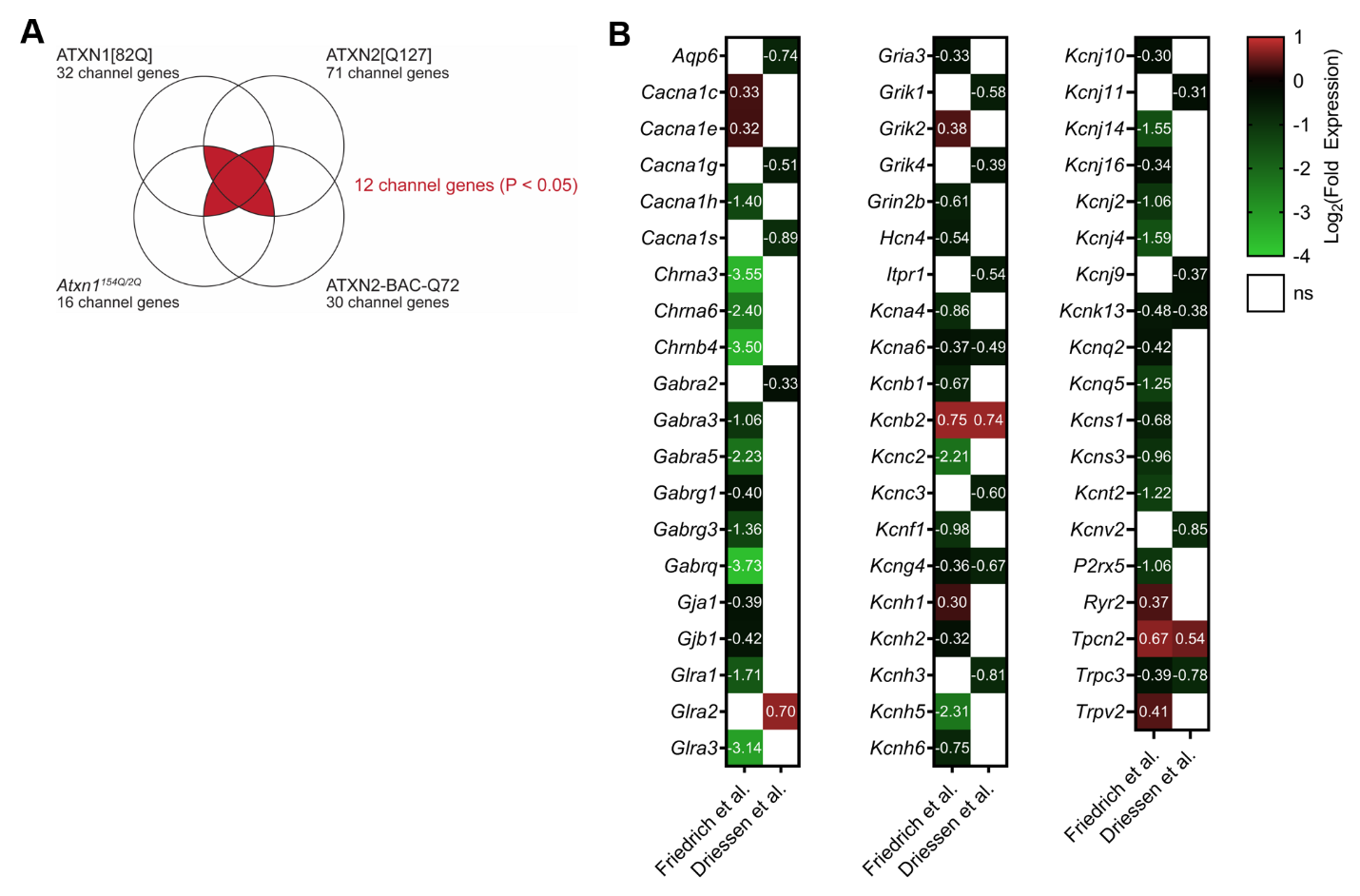


**Supplementary Figure 2. Ion channel transcripts showing dysregulated expression in mouse models of SCA1 and SCA2**

(A) Dysregulated ion channel genes that are found to be shared across whole cerebellar gene expression datasets in SCA1 and SCA2 mouse models. The red overlap represents ion channel genes that are dysregulated in either three or four models. P-values reflect the likelihood of an equivalent number of channels (or more) being dysregulated in any three and in all four models by chance (see methods section). (B) Differentially-expressed ion channel genes from two RNA sequencing studies performed in the *Atxn1^154Q/2Q^* model of SCA1, one performed by Friedrich and colleagues (20), while the other was performed by Driessen and colleagues (19). Log2 transformation of fold change expression is noted for each comparison that achieved statistical significance


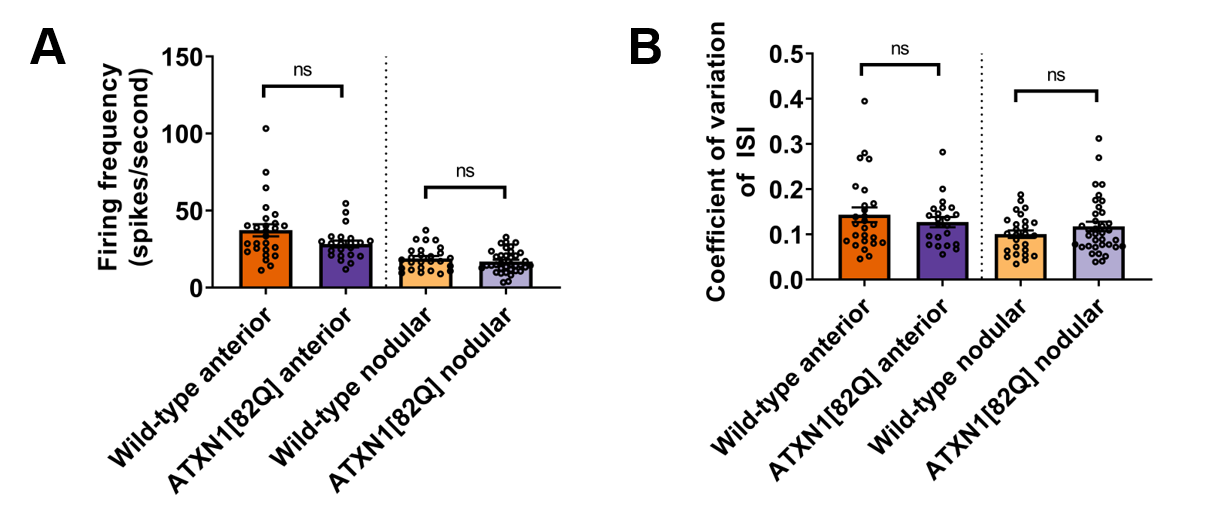


**Supplementary Figure 3. Spontaneous Purkinje neuron firing in ATXN1[82Q] mice and wild-type controls**

Patch clamp electrophysiology in acute cerebellar slices from ATXN1[82Q] mice and wild-type controls was performed at P35. (A) Firing frequency in anterior cerebellum and the nodular zone is shown. (B) Coefficient of variation (CV) of the interspike interval (ISI) is shown for Purkinje neurons in the anterior cerebellum and nodular zone. ns denotes p>0.05; two-tailed Student’s t-test.


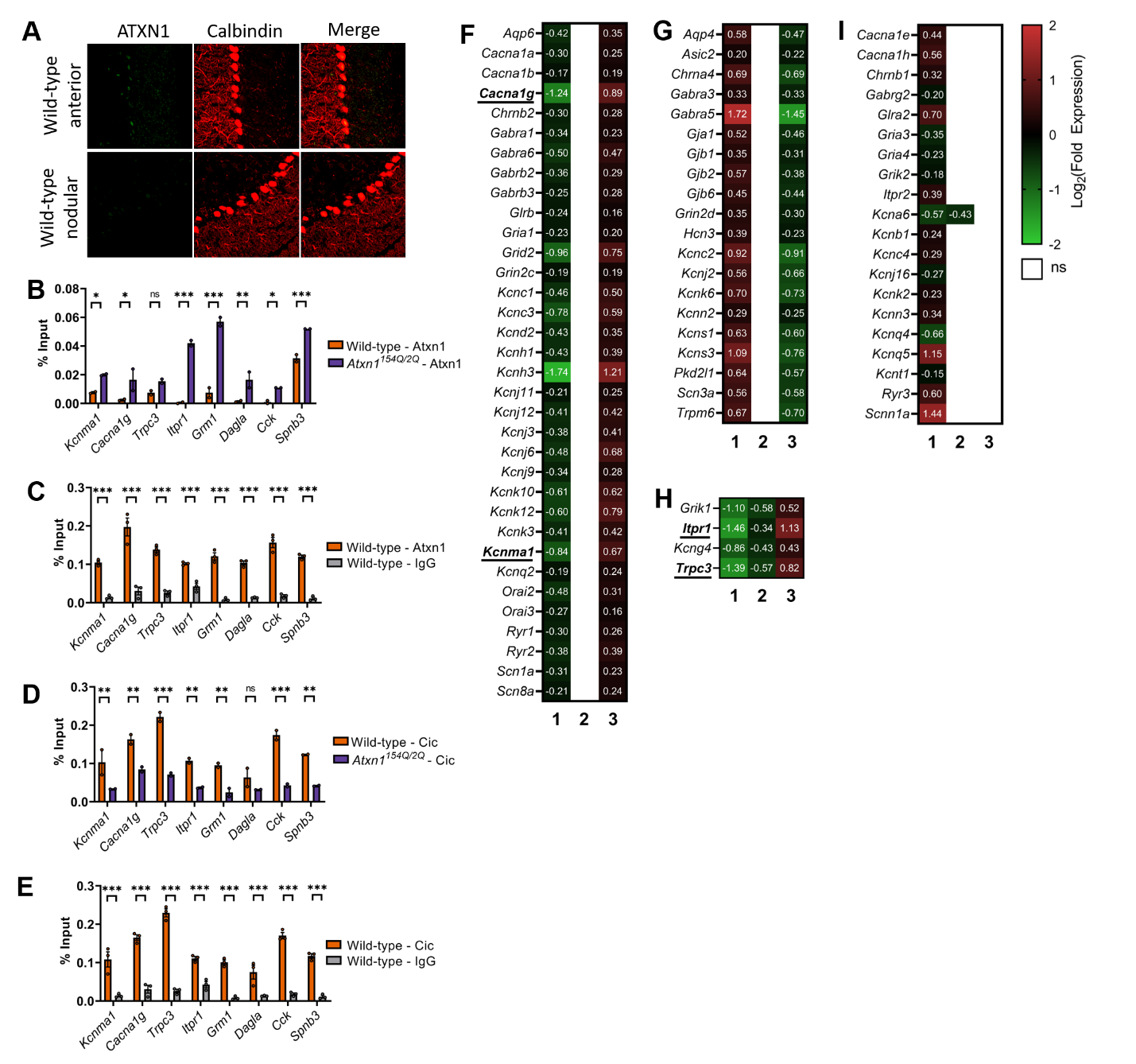


**Supplementary Figure 4. Enhanced binding of Atxn1 and Capicua to the promoter regions of ion channel module genes**

(A) Representative confocal images taken from wild-type mice at P35 after immunostaining for ATXN1 (green) and calbindin (red, to mark Purkinje neurons). (B-E) Quantitative Chromatin immunoprecipitation (qChIP) demonstrating the association of Cic and Atxn1 at the promoter of ion channel genes from sonicated chromatin derived from P14 whole cerebellar extracts. Binding, represented as % input (Y-axis) demonstrated for Atxn1 and Cic comparing their relative binding on ion channel genes in *Atxn1^154Q/2Q^* mice and wild-type controls (B and D, respectively) and binding over background relative to their respective isotype control IgG (rabbit IgG) (C and E, respectively). (F-I) Differential gene expression analysis demonstrating the role for the ATXN1/Cic complex in dysregulation in ion channel genes. Log2 transformation of fold change expression is shown for channel genes that are differentially expressed in ATXN1[82Q] relative to wild-type mice. Each column represents a distinct comparison (numbered by column): 1. ATXN1[82Q] relative to wild-type, 2. ATXN1[82Q]V591A;S602D relative to wild-type and 3. ATXN1[82Q]V591A;S602D relative to ATXN1[82Q]. Data are further subdivided into genes that are downregulated in ATXN1[82Q] and ATXN1/Cic complex dependent (F), upregulated in ATXN1[82Q] and ATXN1/Cic complex depenedent (G), ATXN1/Cic complex partially dependent (H), and ATXN1/Cic complex independent (I). Log2 transformation of fold change expression is noted for each comparison that achieved statistical significance. * denotes p<0.05; ** denotes p<0.01; *** denotes p<0.001; ns denotes p>0.05; two-tailed Student’s t-test with Holm-Sidak correction for multiple comparisons.


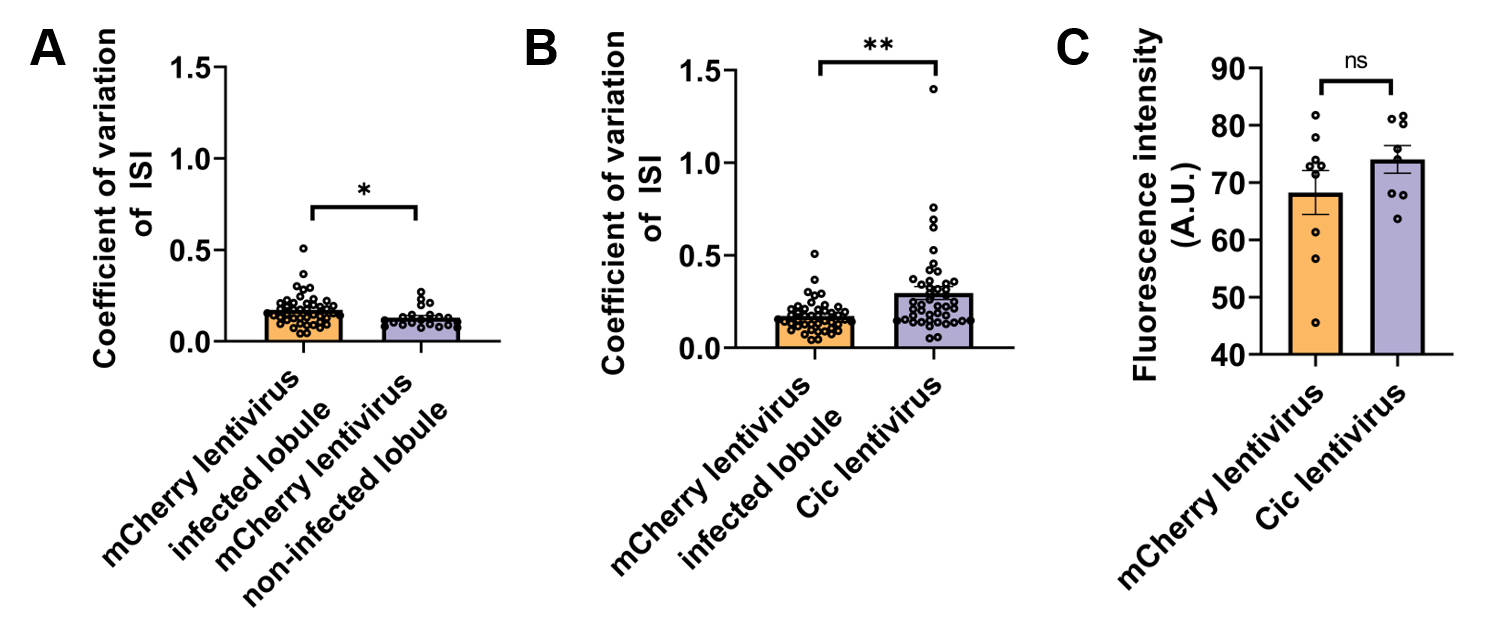


**Supplementary Figure 5. Spontaneous Purkinje neuron spiking after ectopic lentiviral expression of Capicua**

Patch clamp electrophysiology in acute cerebellar slices was performed in the nodular zone of ATXN1[82Q] mice 10 days after lentivirus injection. (A) Coefficient of variation (CV) of the interspike interval (ISI) of Purkinje neuron spiking in the transduced area (lobule IX) and non-transduced area (lobule X) of ATXN1[82Q] mice injected with mCherry lentivirus. (B) CV of the ISI of Purkinje neuron spiking in the transduced area (lobule IX) of ATXN1[82Q] cerebellum for mice injected with either mCherry lentivirus or Cic lentivirus. (C) Cic expression by immunohistochemistry in the transduced area (lobule IX) of ATXN1[82Q] cerebellum from mice injected with either mCherry lentivirus or Cic lentivirus. * denotes p<0.05; ** denotes p<0.01; two-tailed Student’s t-test.
